# Health Consequence Scales for Use in Health Impact Assessments of Climate Change

**DOI:** 10.3390/ijerph110909607

**Published:** 2014-09-16

**Authors:** Helen Brown, Jeffery Spickett

**Affiliations:** School of Public Health, World Health Organisation Collaborating Centre for Environmental Health Impact Assessment, GPO Box U1987, Perth, WA 6845, Australia; E-Mail: j.spickett@curtin.edu.au

**Keywords:** climate adaptation, health impact assessment, health consequence scales

## Abstract

While health impact assessment (HIA) has typically been applied to projects, plans or policies, it has significant potential with regard to strategic considerations of major health issues facing society such as climate change. Given the complexity of climate change, assessing health impacts presents new challenges that may require different approaches compared to traditional applications of HIA. This research focuses on the development of health consequence scales suited to assessing and comparing health effects associated with climate change and applied within a HIA framework. This assists in setting priorities for adaptation plans to minimize the public health impacts of climate change. The scales presented in this paper were initially developed for a HIA of climate change in Perth in 2050, but they can be applied across spatial and temporal scales. The design is based on a health effects pyramid with health measures expressed in orders of magnitude and linked to baseline population and health data. The health consequence measures are combined with a measure of likelihood to determine the level of risk associated with each health potential health impact. In addition, a simple visual framework that can be used to collate, compare and communicate the level of health risks associated with climate change has been developed.

## 1. Introduction

Health Impact Assessment (HIA) has been identified as a valuable tool for systematically identifying, and where possible, quantifying the multiple pathways that link climate change to human health [1,2,3,4,5,6]. There are many challenges in assessing health impacts of climate change, yet estimates of impacts are essential to inform policy discussion on both climate change mitigation and adaptation [3]. The United Nations’ Framework Convention on Climate Change (1992) deems that national governments have a responsibility to carry out formal assessments of the risk to their population’s health posed by global climate change [7]. Health Risk Assessments (HRA) conducted within a HIA framework can meet this responsibility. 

Traditional risk assessment combines a measure of likelihood and consequences, based on predetermined criteria, to estimate a level of risk. The use of predetermined criteria enables a comparison of risk associated with dissimilar effects [8]. This comparative process is critical when determining priorities for research and policy actions. Climate change assessments typically compare the expected health impacts under one or more climate change scenarios with a future scenario of no-climate change, or unavoidable climate change [9]. Because assessments of climate change usually relate to future exposures, the impacts are modelled rather than measured [10]. 

The Intergovernmental Panel on Climate Change (IPCC) has published likelihood scales that can be applied across a wide array of sectors such as health, business or agriculture. The level of likelihood of a well-defined outcome is determined on the basis of quantitative analyses or an elicitation of expert views [11]. In contrast, the vastly different consequences that occur in different sectors prohibit the use of a shared scale for consequences. A determination of the severity of consequences is also influenced by the perception of risk of the affected population. 

While various health impact assessments have been conducted on a range of potential impacts of climate change, few have employed predetermined criteria of health consequences and likelihoods that enable a comparison of risk. For example, the US National Assessment of the Potential Consequences of Climate Variability and Change judges whether a specific health impact is likely to increase, decrease or change in either direction [12]. Health Canada identifies and describes variables that contribute to vulnerability for a range of health impacts, but do not provide estimates of future risks using clearly defined criteria [13]. Various assessments in Australia have estimated health impacts of climate change with respect to increased temperature and heatwaves, flooding, food poisoning and dengue, but these do not use a common health consequence scale nor are they described in terms of risk levels [14,15].

## 2. HIA of Climate Change in Perth—Health Consequence Scales 

The aim of this research was to develop a health consequence scale that could be applied to a HIA of climate change in Perth, Western Australia (WA). The research was part of a national study into climate change adaptation in urban areas of Australia [16].

The development of the health consequence scales began with a review of three key sources:

- HIA of climate change: Adaptation strategies for WA [3]
- Health sector report of the UK Climate Change Risk Assessment (UKCCRA) [17]
- HRA (Scoping) Guidelines [18]

A ‘Health Impact Assessment of climate change: Adaptation strategies for Western Australia’ was a state-wide assessment which covered rural and regional areas, as well as urban areas such as the capital city of Perth. A qualitative scale with five health consequence levels ranging from insignificant to catastrophic was developed as part of the study. Given the HIA was conducted in conjunction with over 70 experts in WA, it was considered of particular relevance to the planned HIA of urban areas in Perth. The health consequence scale included descriptive measures of fatalities, hospitalizations and less severe health impacts including impacts on quality of life and delivery of essential goods and services. The report concluded that while the outcomes provided a strong starting position, future assessments should include a more comprehensive and quantitative assessment of health impacts [3]. While quantifying outcomes in situations with high levels of complexity can be difficult, efforts to quantify will typically lead to new insights that progress knowledge in the area. 

One source that reported quantitative and qualitative measures of health impacts of climate change is the climate change risk assessment for the health sector conducted as part of the UKCCRA [17]. The scales include three consequence levels (*i.e.*, low, medium and high) for economic, environmental and social impacts. Health impacts are a subset of the social category and include similar measures to the WA scale: fatalities, ‘harmed’ (measured as hospitalizations in all examples) and a less severe ‘affected’ measure. Each of these metrics is defined as an order of magnitude range such as ‘hundreds of fatalities’ or ‘thousands harmed’. However, as the metrics in the UK scale were developed for a population of 62 million, direct application to Perth or other locations with significantly different population size is not appropriate. 

While order of magnitude scales lack the precision of typical linear scales, they are more appropriate in situations where the level of risk for different hazards is not well understood and likely to span more than one order of magnitude [19]. In addition to the uncertain climate conditions in the future, assessment of the potential health impacts of climate change introduces other uncertainties and it is likely that many assessments of health consequences will span more than one order of magnitude. It was therefore considered that the design of the UK scales, with respect to the order of magnitude health measures, could inform the development of health consequence scales appropriate for Perth. 

The third source was developed in collaboration with local health experts for application to potential health impacts of planned developments and activities in WA [18]. Although the process was not developed for wide-ranging events such as climate change, the quantitative health consequence scales provided guidance with respect to the type of health measures considered appropriate in WA for industrial development projects. The health measures were similar to the other sources, with mortality, hospitalizations and a broad category of less severe health impacts. The quantitative measures for hospitalizations were expressed in terms of the percentage of the population at risk such as ‘more than 5–10% of the population at risk experience an acute health effect requiring hospitalization’. Unlike discrete developments with limited population exposure, climate-related exposures such as extreme heat, air pollutants and food-borne diseases, will occur on a broader population scale. For this reason, the application of the ranges for development projects is not suitable for climate change. Nevertheless, the concept of a clearly defined range expressed in terms of percentage of exposed population, is a feature that was used to inform the development of health consequence scales in this research. 

Although the health consequence scales were initially developed for a HIA of climate change in Perth in 2050, the issues raised during the review of the three key sources led to the possibility of designing a scale that could be applied across time and space. This is particularly important for climate change assessments, as they are likely to span long time periods and be conducted in regional, urban and rural locations in many countries. The ability to compile and compare results from different studies would be a useful way to consolidate such findings to inform regional or national assessments. 

To achieve this it was determined that the health consequence scale should:

- Be linked to population size and baseline health data
- Provide appropriate quantitative and qualitative measures of health outcomes that reflect the range of potential health impacts of climate change
- Be relatively simple and easy to communicate

## 3. Methods

A health consequence scale which meets the above characteristics was developed by utilizing aspects from each of the sources discussed. The link to population size and baseline health data was achieved by making use of data collected as part of the profiling phase of the HIA of climate change in Perth. Likelihood scales were based on the IPCC [11] and standard risk assessment matrices. The initial scales were sent for comment to people with at least ten years’ experience in the field of health risk assessment or health impact assessment. A total of 12 experts provided comments, including authors of the three key sources and representatives from Australian and overseas universities, State government health department staff and risk assessment consultants. Given the specialized nature of assessing health risks associated with climate change, it was considered that the experts’ comments provided sound feedback regarding the scales prior to application in the planned HIA approach. 

The experts were provided with the background and rationale for the scales and asked the following questions:

- Are the proposed health metrics reasonable?
- Are the quantitative ranges used for each health metric reasonable?
- Are the likelihood scales appropriate?
- Does the risk assessment matrix provide a reasonable measure of risk?

Refinements were made to the proposed scales on the basis of reviewer’s comments. The final scales were then applied to Perth for the year 2011 and 2050. 

## 4. Results

An explanation of the final health consequence scale is divided into three key aspects: the selection of health metrics; the relative magnitude of each health consequence level; and the numerical ranges for each health metric at each consequence level. 

### 4.1. Health Metrics 

The proposed health metrics are:

- Total mortality (percentage increase)
- Hospitalizations (percentage increase in potentially preventable hospitalizations)
- Population affected (percentage)

**Figure 1:**
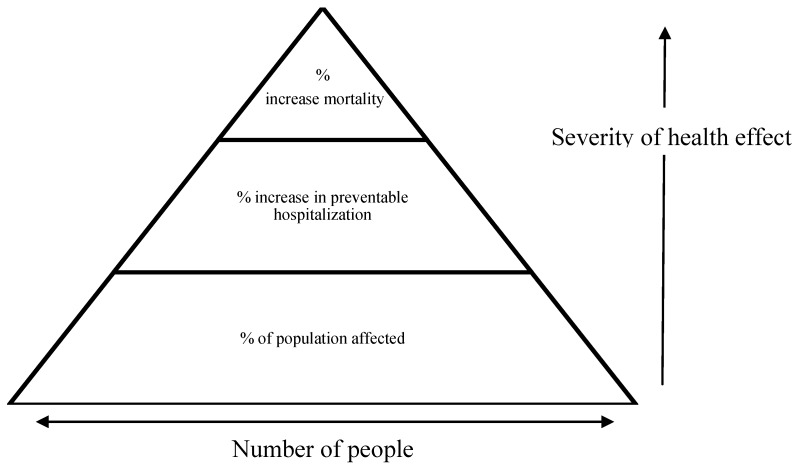
Health effects pyramid.

The metrics can be represented by a health effects pyramid (Figure 1), where the most common, but least severe impacts are shown at the bottom of the pyramid, with progressively less people experiencing more severe health impacts toward the tip of the pyramid. Existing data sources can be used for mortality and hospitalization metrics. Potentially preventable hospitalization (PPH) was selected because the ratio between PPH and total mortality in Perth was similar to the ratio between mortality and hospitalization used in the UKCCRA scales. In addition the definition of PPH was considered to cover the range of potential hospitalizations likely to occur as a result of climate change in Perth [20]. The third metric is a composite of measures including: health effects not requiring hospitalization; significant decline in delivery of essential goods and services; and significant long-term reductions in quality of life. 

### 4.2. Relative Magnitude of Each Health Consequence Level

**Figure 2:**
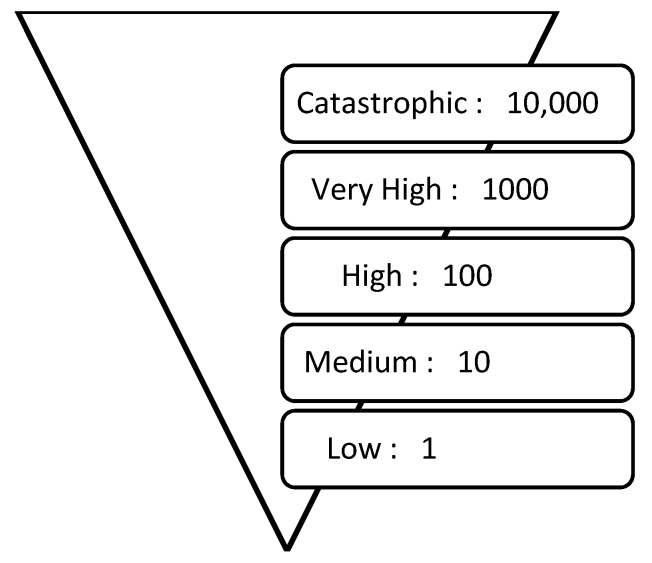
Ratio of health metrics across health consequence levels.

The second aspect of the scale is the ratio between health consequence levels. The UKCCRA used a 1:10 ratio between each level and this was considered appropriate for the proposed scales. While the UK scales included three consequence levels, the final scales have five levels, as used in both of the WA sources. Figure 2 shows the ratio of health consequence levels, beginning with an arbitrary baseline of 1 for ‘Low’ and increasing by a set factor of 10 at each level. For example, ‘Very High’ is a health consequence which is 100 times greater than a ‘Medium’ health consequence.

Each health consequence level will be determined on the basis of one or more of the metrics in the health effects pyramid. For example, a catastrophic health consequence does not necessarily involve any fatalities or hospitalizations—if a sufficient number of people are affected in less severe ways, this can still be considered as catastrophic. It is likely that climate-related impacts for a single hazard such as heatwaves will include fatalities, hospitalizations and the less severe ‘affected’ variable. 

### 4.3. Numerical Range for Each Health Consequence Level

The first two aspects determined the overall design of the scale. The final aspect is the numerical range of each health consequence level. The UKCCRA scales were the only scales with quantitative measures developed specifically for health impacts of climate change and these figures were considered an appropriate starting point. Adjustments based on population size and differences in mortality rate were made by converting the UKCCRA measures into percentage increase in mortality. 

The high consequence level, with respect to health, in the UKCCRA was ‘hundreds of fatalities’. With approximately 500,000 deaths per annum in the UK [17], an increase of 100 fatalities represents an increase of approximately 0.02% in total mortality. This figure was used as a starting point to define the numerical ranges. The 1:10 ratio as shown in Figure 2 was used to define each health consequence level in terms of percentage increases in mortality. For example, the catastrophic level, which is 100 times greater than the high level, was defined as ‘greater than a 2% increase in mortality’. 

With the mortality levels determined, the next step was to determine the hospitalization and affected levels of the health effects pyramid. The UK scale employed a fixed ratio of one death per ten hospitalizations per 10,000 people affected. However, because it had been determined that the scales will be linked to demographic and health data, a fixed ratio will not be a feature of the scales. Nevertheless, the ratios were used to guide the selection of appropriate measures. Analysis of Perth metropolitan health data indicated that the number of PPH was approximately 5 times greater than total annual deaths [21]. To achieve a ratio of approximately 1:10 the percentage increase in PPH was set at double the percentage increase in mortality. Testing these figures, a 2% increase in mortality for a Perth population of 1.7 million, is estimated at 195 deaths and a 4% increase in PPH estimated at 1830 hospitalizations. This represents a ratio 1 to 9.4—slightly lower than the 1 to 10 ratio in the UK scale, but still considered suitable for the proposed scale. 

The final affected metric is a composite measure of health-related outcomes selected from the reviewed scales. The components of the affected metric are shown in full in Table 1. These effects are likely to vary considerably, depending on the health impact being assessed, and expert judgment will be required to make appropriate estimates. Applying the UK ratio, the minimum number of affected people in Perth in 2011 at the ‘Very High’ consequence level was 183,000 people, which is 10.8% of the population. Bearing in mind, the requirement for a scale that is relatively simple to communicate, it was determined that this figure be amended to 10%.

**Table 1:** Health consequence scales applied to Perth in 2011 and 2050.

Level	Mortality Increase (%)	PPH Increase (%)	Population Affected * (%)	Results as applied to Perth in the year:	
				2011	2050
Catastrophic	>2	>4	>90	>195 deaths >1830 PPH >1.5 million affected	>356 deaths >3340 PPH >2.8 million affected
Very High	>0.2–2	>0.4–4	>10–90	>20 deaths >183 PPH >170,000 affected	>36 deaths >334 PPH >280,000 affected
High	>0.02–0.2	>0.04–0.4	>1–10	>2 deaths >18 PPH >17,000 affected	>4 deaths >34 PPH >28,000 affected
Medium	>0.002–0.02	>0.004–0.04	>0.1–1	≤2 deaths >2 PPH >1700 affected	>0 deaths >3 PPH >2800 affected
Low	≤0.002	≤0.004	≤0.1	0 deaths ≤2 PPH ≤1700 affected	0 deaths ≤3 PPH ≤2800 affected

* Health effects not requiring hospitalization; significant decline in delivery of essential goods and services; significant long-term reductions in quality of life.

The expert reviewers suggested several limitations of the proposed scales. The first limitation relates to population size and it is proposed that the scales are only practical for a population of at least 1 million. The affected measure for the catastrophic consequence was adjusted from 100% to 90% as this was deemed a more useful measure. In addition, the projection of health metric ranges in 2050 only considered changes in population size. There will clearly be other factors such as an ageing population that will influence the baseline rate of mortality and hospitalizations; however it was considered that a significant proportion of these influences would be captured by the order of magnitude scales. In addition, we return to the argument of providing a balance between accuracy and practicality and the ultimate aim of the process—to provide a comparative measure of risk that can help to inform decision-making in policy and research. 

### 4.4. Application of Health Consequence Scale to Perth

The final step was to apply the health consequence scale to a Perth population of 1.7 million for the year 2011 and a population of 3.1 million for the year 2050 [22]. The most recent 5-year figures (2005 to 2009) on mortality and PPH rates in Perth were adjusted for population size to estimate the expected number of annual deaths (9758) and PPH (45,772) in Perth in 2011 [21].The expected number of deaths and hospitalizations in 2050 was adjusted on the basis of projected population increases. The full set of results is shown in Table 1. 

The proposed scale defines a ‘Very High’ health consequence in Perth in 2011 as: between 20 and 195 deaths; or between 183 and 1830 PPH; or more than 170,000 people affected. To provide context, the number of deaths due to road accidents in Perth in 2012 was 74 [23] and the number of hospitalizations due to road traffic injuries in Perth was approximately 1400 [24]. Both of these figures fall within the ‘Very High’ health consequence level of Table 1. It was considered reasonable that an impact of the same order of magnitude of the annual road toll would be considered a ‘Very High’ health consequence. Extending this rationale, the number of deaths that result in a catastrophic health ranking is equivalent to at least 3 times the annual road toll deaths recorded in metropolitan Perth in 2011.

### 4.5. Determination of Risk

The above health consequence scale is used in conjunction with the likelihood scale in Table 2. The scale is a slight adjustment of the IPCC likelihood scale [11] with the two extreme levels of <1% and >99% removed. It is unlikely that health-related outcomes included in climate change assessments will have likelihoods of <1% or >99% at this time. It was also considered that five levels for likelihood resulted in a more practical risk assessment matrix.

**Table 2:** Likelihood scale.

Level	Likelihood of the occurrence/outcome
Very Unlikely	<10%
Unlikely	<33%
About as likely as not	33–66%
Likely	>66%
Very Likely	>90%

The occurrence or outcome will be defined by the health consequence scale. The HIA of climate change in Perth concluded that between 36 and 356 additional heat-related deaths would occur in Perth in 2050 as result of climate change. Referring to Table 1, this is considered a ‘Very High’ health consequence. The ability to judge likelihood of outcomes expressed in this way is far greater than determining the likelihood of narrow outcomes. The likelihood of between 36 and 356 heat-related deaths, based on a consideration of the evidence, was judged as >90% or ‘Very Likely’.

The decision was made to enter the results from the health consequence and likelihood assessments into a standard risk assessment matrix as shown in Table 3. This results in a level of risk for each potential health impact ranging from ‘Very Low’ to ‘Extreme’. This format is: (i) widely accepted across a range of settings, (ii) familiar to people with knowledge of risk assessment and (iii) relatively simple to communicate. 

**Table 3:** Risk assessment matrix and final risk levels.

Likelihood	Health Consequence				
	Low	Medium	High	Very High	Catastrophic
Very Unlikely	Very Low	Very Low	Low	Low	Medium
Unlikely	Very Low	Low	Low	Medium	High
About as likely as not	Low	Low	Medium	High	Very High
Likely	Low	Medium	High	Very High	Extreme
Very Likely	Medium	High	Very High	Extreme	Extreme

The ‘Very High’ health consequence for heat-related mortality, which was deemed ‘Very Likely’, resulted in a final risk level of ‘Extreme’. Each level of risk can occur via different combinations of health consequence and likelihood. For example, as shown in Table 3, a ‘Very High’ risk level can occur as a result of the following *likelihood* and health consequence levels: (i) *About as likely as not* and Catastrophic; (ii) *Likely* and Very High; and (iii) *Very Likely* and High. 

## 5. Discussion

The capacity to compare the risk levels associated with different health impacts is an essential part of HIA. That capacity is aided by a clear set of pre-determined measures regarding the severity and likelihood of health consequences resulting from climate change. Despite the challenges of conducting a quantitative assessment of health impacts of climate change, the advantages are clear. In the first instance, a clearly defined set of measures ensures a more objective measure of risk than qualitative scales. Secondly it highlights potential shortcomings in the available evidence. For example, application of the scales in Perth concluded that there was insufficient evidence for an order of magnitude estimate for health impacts related to aeroallergens and vector-borne diseases. The previous state-wide HIA of climate change, which used qualitative health consequence scales, determined each of these impacts as a high risk level. The order of magnitude health metrics compelled a more critical analysis of the available data and raised important questions on how limitations in evidence can be addressed. 

The third advantage offered by the scales is that because they are normalized against population and health data they can be applied to other locations and time periods. This offers a standardized approach that helps to consolidate and compare information from a vast pool of evidence. A thorough investigation of population and health data in other countries was beyond the scope of this research and while it is acknowledged that some adjustments may be necessary, it is proposed that the basic design of the health effects pyramid will be appropriate for most locations. 

**Figure 3:**
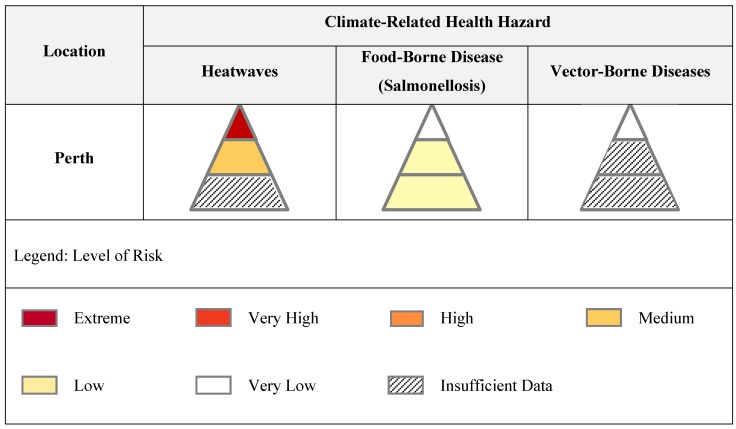
Matrix of health risks of climate change.

The final advantage is that the outcomes of the process can be communicated in a relatively simple manner. The ability to present complex information in a format that is relevant and useful to policy makers is critical [10]. Figure 3 depicts the results of the process as applied to three climate-related health hazards in Perth. This can be expanded to any location with a population of at least one million and across the full-range of climate-related hazards.

Figure 3 indicates that the assessment in Perth of the health risks of heatwaves associated with climate change was determined as:

- ‘Extreme’ with respect to mortality
- ‘Medium’ with respect to hospitalizations
- Undetermined level of risk with respect to the percentage of population affected

In cases where the levels of risk associated with each layer of the health effects pyramid differ, the highest level of risk takes precedence. The overall level of risk of heatwaves associated with climate change in Perth was therefore determined as ‘Extreme’. In addition, Perth had a ‘Low’ level of risk assigned to the PPH and affected health metrics for Salmonellosis and a ‘Very Low’ level of risk with respect to the mortality metric. There were a number of metrics, including those shown in the vector-borne disease pyramid, where a determination of risk using the predetermined health consequence and likelihood scales was not possible based on the available evidence. Further details of the assessment in Perth will be provided in a forthcoming manuscript. 

This matrix provides a simple visual method of aggregating information, identifying gaps in current knowledge and communicating potential health risks. Links to more detailed information can be provided for each segment of the health effects pyramid. These results can be used to direct research programs as well as climate change adaptation strategies. A conclusion of ‘inadequate data’ for an impact will raise obvious questions of whether the gap can be filled by analysis of current evidence or new research. For example, the UKCCRA originally excluded injuries from flooding because they were considered too difficult to assess. However a subsequent review of new data indicated a relatively simple relationship between flood-related mortality and injuries, thereby enabling an assessment [17]. Even if the ‘gap’ is unlikely to be filled due to methodological limitations, it is important to acknowledge this. 

Considering potential adaptation strategies for each segment of every health effects pyramid can help to identify potential synergies and/or conflicts between adaptation strategies. In addition, the identification of key stakeholders involved in each segment would raise awareness of adaptation where cross-sectoral collaboration and understanding is critical. 

A significant proportion of published assessments report health impacts of climate change in terms of fatalities or hospitalizations. While this is understandable in terms of data collection, the health effects pyramid serves as a reminder that impacts should be considered across all levels. If assessments focus primarily on fatalities, less severe impacts that nevertheless have the potential to impact significantly on health and well-being may be overlooked. This may prove particularly important in countries such as Australia, where high adaptive capacity and strong health-care may lead to relatively few cases of the most severe impacts. 

Finally, since a HRA of climate change requires an assessment of the expected health impacts without climate change, the framework provides an important assessment of existing levels of risk. This may prove especially useful for identifying and communicating ‘low-regrets’ policy options which will improve health outcomes, regardless of future climate outcomes. 

## 6. Conclusions

The application of a HIA framework to broad health issues of strategic importance, such as climate change, represents a shift from the typical application of HIA to projects, plans or policies. This shift is likely to require new or amended tools used within a HIA framework. The health consequence scales and matrix for climate-related health risks presented in this paper are examples of tools that may be suited to HIAs of climate change. 

Given the unique challenges of climate change and the relative infancy of research in assessing health impacts of climate change, it should be recognized that the process of developing appropriate health consequence scales is highly iterative. As the evidence and certainty regarding health impacts of climate change increases, the order of magnitude ranges could be amended to narrower ranges. In addition, application of the scales will be likely to lead to refinements of the mortality, hospitalization and affected health measures presented here. For example, as more evidence is collected with respect to the broad ‘population affected’ metric, more specific measures regarding particular diseases or conditions linked to climate-related health risks, can be developed. 

Effective scales need to provide a balance between accuracy, practicality and the ability to provide information that can communicate risk effectively and inform decision-making. At this stage, we consider the order of magnitude estimates for health consequences and the likelihood scales are commensurate with the current level of knowledge.

The application of predetermined health consequence scales within a HIA framework enables prioritization of suitable actions to address climate change. It also highlights situations where the current level of evidence may be insufficient to determine even order of magnitude estimates. Identification of such instances is an important step in planning appropriate research and ensuring that adaptation planning is based on the best available evidence. It is hoped that further application will assist in the use of a HIA framework for strategically important health issues such as climate change and will provide suggestions for further refinement of the process.
